# Association of vitamin D metabolites with cognitive function and brain atrophy in elderly individuals - the Austrian stroke prevention study

**DOI:** 10.18632/aging.202930

**Published:** 2021-04-07

**Authors:** Sieglinde Zelzer, Edith Hofer, Andreas Meinitzer, Eva Fritz-Petrin, Sebastian Simstich, Walter Goessler, Reinhold Schmidt, Markus Herrmann

**Affiliations:** 1 Clinical Institute of Medical and Chemical Laboratory Diagnostics, Medical University of Graz, Austria; 2 Clinical Division of Neurogeriatrics, Department of Neurology, Medical University of Graz, Austria; 3 Institute for Medical Informatics, Statistics and Documentation, Medical University of Graz, Austria; 4 Institute of Chemistry, University of Graz, Austria

**Keywords:** vitamin D metabolites, LC-MS/MS, cognitive function, memory, dementia

## Abstract

Background: Vitamin D is a well-established regulator of calcium and phosphate metabolism that has neurotrophic and neuroprotective properties. Deficiency of vitamin D has been proposed to promote cognitive dysfunction and brain atrophy. However, existing studies provide inconsistent results. Here we aimed to investigate the association between vitamin D metabolites, cognitive function and brain atrophy in a cohort of well-characterized community-dwelling elderly individuals with normal neurological status and without history of stroke and dementia.

Methods: 25(OH)D_3_, 25(OH)D_2_ and 24,25(OH)_2_D_3_ were measured by liquid-chromatography tandem mass-spectrometry in serum samples from 390 community-dwelling elderly individuals. All participants underwent thorough neuropsychiatric tests capturing memory, executive function and visuopractical skills. In 139 of these individuals, MRI of the brain was performed in order to capture neurodegenerative and vascular changes.

Results: Total 25(OH)D (ß=0.003, 0.037), 24,25(OH)_2_D_3_ (ß=0.0456, p=0.010) and vitamin D metabolite ratio (VMR) (ß=0.0467, p=0.012) were significantly related to memory function. Adjustment for multiple testing weakened these relationships, but trends (p≤0.10) remained. 24,25(OH)_2_D_3_ and VMR showed similar trends also for visuopractical skills and global cognitive function. No significant relationships existed between vitamin D metabolites and MRI derived indices of neurodegeneration and vascular changes. Sub-group analyses of individuals with low concentrations of 25(OH)D and 24,25(OH)_2_D_3_ showed significantly worse memory function compared to individuals with normal or high concentrations.

Conclusions: Vitamin D deficient individuals appear to have a modest reduction of memory function without structural brain atrophy. Future studies should explore if vitamin D supplementation can improve cognitive function.

## INTRODUCTION

Dementia, and in particular Alzheimer’s disease (AD), is a major cause of disability and dependency among older people worldwide [1]. It has been estimated that in 2018 approximately 50 million people were affected by AD and this number is expected to rise to an estimated 152 million by 2050 [2]. The disease is characterized by a progressive decline in cognitive function that is commonly accompanied, and occasionally preceded, by a deterioration of emotional control, social behaviour, or motivation [3].

At present, curative therapies are lacking and preventive measures that potentially delay onset and progression of the disease are of particular importance. Factors known to lower the incidence of Alzheimerʹs disease are educational attainment, social integration, regular physical activity, and treatment of vascular risk factors at midlife [4]. An adequate supply with vitamin D, a well-established regulator of calcium and phosphate metabolism, immune function, cell proliferation and differentiation, has been proposed to delay cognitive decline and brain atrophy [4, 5]. If true, this would be of great public interest as vitamin D deficiency is a highly prevalent condition in developed countries that can easily be corrected by UV-irradiation of the skin or oral vitamin D supplementation [6, 7].

Several lines of evidence indicate that vitamin D has neurotrophic and neuroprotective properties, and is involved in brain development [5]. The vitamin D receptor (VDR) is widely present in the brain with highest expression in the hippocampus, hypothalamus, thalamus, cortex, subcortex and substantia nigra [8, 9]. The same areas, which are related to cognitive function, also express 1α-hydroxylase, an enzyme that converts inactive 25-hydroxyvitamin D (25(OH)D) into active 1,25-dihydroxy vitamin D (1,25(OH)_2_D). Besides the intracerebral synthesis of 1,25(OH)_2_D, vitamin D metabolites can enter the brain by crossing the blood-brain barrier [10]. In the brain, vitamin D upregulates neurotrophic factors, such as nerve growth factor (NGF), glial-derived nerve growth factor (GDNF), and neurotrophin 3 (NTF3) [11] and exerts neuroprotective effects [5]. Furthermore, vitamin D supplementation seems to improve cholinergic function through reduction of oxidative stress and neuroinflammation [12, 13]. Based on these observations it has been speculated that vitamin D deficiency might promote cognitive decline and thus increase the incidence and progression of AD [14]. While some cross-sectional and longitudinal studies support this hypothesis [15, 16], others do not [17–19]. So far, only a handful of studies investigated the relationship between serum 25(OH)D and brain atrophy with mixed results [20, 21].

In line with current recommendations, all existing studies assessed vitamin D status by measuring serum 25(OH)D. 24,25(OH)_2_D is the catabolite of 25(OH)D that is only formed through the action of 24-hydroxylase (CYP24A1) when sufficient amounts of 25(OH)D are available. The simultaneous analysis of 24,25(OH)_2_D_3_ may be helpful in identifying individuals with functional vitamin D deficiency or defects of 24-hydroxylase [22, 23]. Low or undetectable concentrations of 24,25(OH)_2_D_3_ might indicate CYP24A1 deficiency or functional vitamin D deficiency where all 25(OH)D is needed to maintain an adequate cellular supply with active 1,25(OH)_2_D. Another limitation of previous studies is the measurement of 25(OH)D with different immunoassays, which have shown variable accuracy in comparison to validated LC-MS/MS reference methods [24, 25].

The present study aimed to expand existing knowledge by analysing 24,25(OH)_2_D_3_, 25(OH)D_3_ and 25(OH)D_2_ simultaneously with a validated in-house LC-MS/MS method, calculating the vitamin D metabolite ratio (VMR) [23], and assessing cognitive function and MRI-based brain atrophy in a well-characterized cohort of community-dwelling non-demented elderly Austrians.

## MATERIALS AND METHODS

### Study design

**Figure 1:**
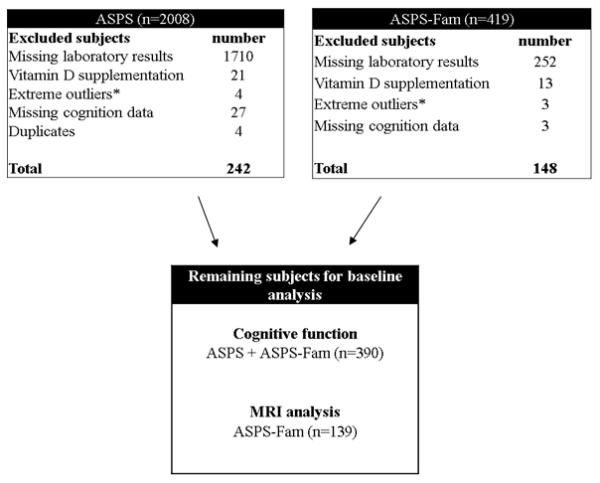
**Flow diagram on patient recruitment.** ASPS: Austrian Stroke Prevention Study, ASPS-Fam: Austrian Stroke Prevention Family Study.*Extreme outliners: mean+3x standard deviation 5.

Measurements of the vitamin D metabolites, 25(OH)D_3_, 25(OH)D_2_ and 24,25(OH)_2_D_3_ were performed in 390 stored serum samples at -80° C from community-dwelling elderly individuals with normal neurological status and without history of stroke and dementia. Neuropsychiatric tests capturing memory, executive function and visuopractical skills were used for analysing cognitive function of all participants. In 139 individuals MRI of the brain was performed in order to capture neurodegenerative and vascular changes. Only subjects with a complete set of biochemical and cognitive test results and without vitamin D supplementation were included in this study. Results were used to explore a potential association between vitamin D status, cognitive function and structural markers of neurodegeneration. The selection of participants for the final study cohort is shown in Figure 1.

### Participants

The study population was assembled by community-dwelling elderly individuals with normal neurological status and without history of stroke and dementia from the Austrian Stroke Prevention Study (ASPS) and the Austrian Stroke Prevention Family Study (ASPS-Fam). The ASPS is a prospective single-centre study examining the effects of vascular risk factors on brain structure and function [26, 27]. Randomly selected individuals from the community register of the city of Graz, Austria, were enrolled between 1991 and 2004. All study protocols were approved by the ethics committee of the Medical University of Graz, Austria, and written informed consent was obtained from all participants.

Inclusion criteria were absence of stroke and dementia, and a normal neurologic examination. ASPS-Fam is an extension of ASPS with a similar study protocol consisting of ASPS participants and their first-grade relatives [26, 27]. Since 3D T1 and FLAIR sequences were not available for the ASPS cohort, the dataset for MRI analyses included 139 individuals from ASPS-Fam permitting automated evaluation of brain atrophy measures.

The risk factors hypertension, diabetes mellitus and atrial fibrillation were examined in all participants. Hypertension was defined as history of hypertension or systolic blood pressure over 140 mmHg or a diastolic blood pressure over 90 mmHg [28] and current use of antihypertensive agents. Subjects were classified as diabetic on the basis of a documented history of diabetes, use of anti-diabetics or a fasting blood glucose level above 126 mg/dl (7.0 mmol/L) at the time of examination [29]. The presence of atrial fibrillation was confirmed by an electrocardiogram obtained during the study visit.

### Measurement of vitamin D metabolites

Vitamin D metabolites were determined in serum samples (50 μl) using a validated in-house liquid-chromatography tandem mass-spectrometry (LC-MS/MS) method for the simultaneous measurement of 25(OH)D_3_, 25(OH)D_2_ and 24,25(OH)_2_D_3_. Details of this method have been reported previously [23]. Briefly, after protein precipitation with potassium hydroxide, vitamin D metabolites were isolated by liquid/liquid extraction with n-heptane:tert-methyl-butyl-ether (1+1) and subsequently derivatized with 4-phenyl-1,2,4-triazoline-3,5-dione (PTAD). d6-25(OH)D_3_, d3-25(OH)D_2_, and d6-24,25(OH)_2_D_3_ were used as internal standards. In the next step, samples were separated on an Agilent HPLC 1260 system using a Kinetex® 5 μm F5 100Å LC Column (150 x 4.6 mm, Phenomenex, Torrance, CA, USA) and a water/acetonitrile gradient. A Sciex 4500 MS/MS instrument was employed for the detection of vitamin D metabolites with a run time of 17 min. Quantitation of the metabolites was performed by direct determination of peak area ratios of 25(OH)D_3_ (m/z = 558.4 / 298, retention time (RT) = 8.35 min), 25(OH)D_2_ (m/z = 570.2 / 298, RT = 8.52 min) and 24,25(OH)_2_D_3_ (m/z = 574.2 / 298, retention time (RT) = 6.24 min) [23]. VMR was calculated as the ratio between 24,25(OH)_2_D_3_/25(OH)D_3_/ x 100 (VMR, %) [30].

### Neuropsychological testing

Cognitive function was assessed with dedicated test batteries. A description of these test batteries has been published previously [27, 31–35]. In order to reduce sources of measurement error, we used composite measures of the cognitive domains memory, executive function and visuopractical skills in the analyses rather than the results of individual tests. These summary measures were calculated by converting test results to z-scores based on the mean and standard deviation of the combined ASPS and ASPS-Fam sample, and by computing the average z-scores within each cognitive domain.

Additionally, principal components analysis was used to calculate a measure of global cognitive ability (g-factor) combining the results from all individual tests [36].

### Magnetic resonance imaging (MRI)

ASPS-Fam participants underwent MRI on a 3T whole-body MR system (TimTrio; Siemens Healthcare, Erlangen, Germany). MRI scans from ASPS participants could not be considered in the present study as they were obtained in the 1990s, when 3D T1 and FLAIR sequences were not yet available.

Total, cortical and subcortical gray matter volume, hippocampus volume and lobar cortical volume, thickness and surface area were computed from the T1 weighted MPRAGE images using FreeSurfer 5.3 [37, 38]. Based on the intensity of the voxels in the MRI image the software automatically segments the brain into subcortical gray volumetric structures and cortical gray matter. Freesurfer divides the cerebral cortex into gyral based regions of interest and provides the cortical volume and thickness for each of these regions. Values of these regions were added up or averaged for volume, surface area, and cortical thickness, respectively, to obtain these measures for the lobes. To correct for variations in individual head size, all measures were normalized for total intracranial volume.

### Statistical analysis

Statistical analysis was performed using the R software version 3.6.1 [39]. We assessed normality of continuous variables by visual inspection and Shapiro-Wilk’s test.

Violin plots showing the distribution of the laboratory parameters were generated using the R package ggplot2 [40]. Normally distributed variables are reported as mean ± standard deviation (STD) and non-normally distributed variables as median and interquartile range (IQR). Demographics, Risk Factors and vitamin D metabolites were compared between ASPS and ASPS-Fam using Chi-Square test for categorical variables and Mann-Whitney-U test for non-normally distributed continuous variables. The Mann-Whitney-U test was also used to compare the vitamin D metabolites between spring and the other three seasons.

We first determined the linear association between vitamin D metabolites and cognition as well as MRI by including the vitamin D metabolites as continuous predictors. Subsequently, we compared the measures of cognition between individuals with and without deficient vitamin D metabolite concentrations. Based on the recommendation of the IOM [41], 25(OH)D deficiency was defined as < 50 nmol/L. In the absence of formally established reference ranges, we set arbitrary cut-offs for 24,25(OH)_2_D_3_ and the VMR on the basis of existing literature [22] and our own experience. These cut-offs were < 3 nmol/L for 24,25(OH)_2_D_3_ and < 3% for the VMR. All analyses were adjusted for age, sex, hypertension, diabetes and atrial fibrillation, and the season of blood draw. Cognition analyses were additionally adjusted for education, and, as we pooled ASPS and ASPS-Fam data for these analyses, we also used the study as covariate to adjust for any undetected differences between the two studies. ASPS-Fam is a family study and therefore we calculated linear mixed models with the family structure as a random effect as implemented in the lmekin function of the R package coxme [42]. A kinship matrix describing the degree of relationship between any two individuals in the study was generated using the R package kinship2 [43]. The results of linear mixed model analyses are presented as regression coefficient (β), standard error of the regression coefficient (SE) and p-values (p). For all p-values within Tables 1, 2 we applied false discovery rate (FDR) correction [44] to compensate for the number of tests in the table.

**Table 1:** Linear relationship between vitamin D metabolites and cognitive function in community-dwelling elderly individuals with normal neurological status and without history of stroke and dementia (ASPS + ASPS-Fam).

**ASPS+ASPS-Fam (n=390)**	**25(OH)D [nmol/L]**				**24,25(OH)_2_D_3_ [nmol/L]**				**VMR [%]**			
	**β**	**se**	**p**	p*	**β**	**se**	**p**	p*	**β**	**se**	**p**	p*
executive function	0.0002	0.001	0.872	0.87	0.0073	0.014	0.593	0.71	0.0064	0.014	0.659	0.72
visuopractical skills	0.0013	0.001	0.339	0.45	0.0351	0.017	0.038	0.10	0.0335	0.018	0.061	0.10
memory	0.0030	0.001	0.037	0.10	0.0456	0.018	0.010	0.07	0.0467	0.019	0.012	0.07
g-factor	0.0019	0.001	0.186	0.28	0.0346	0.017	0.048	0.10	0.0348	0.018	0.059	0.10

All analyses are adjusted for age, sex, hypertension, diabetes, atrial fibrillation, education, study, season and family structure. β: regression coefficient se: standard error of the regression coefficient p*: p-value adjusted for multiple testing (false discovery rate).

ASPS: Austrian Stroke Prevention Study, ASPS-Fam: Austrian Stroke Prevention Family Study, 25(OH)D: sum of 25(OH)D_3_ + 25(OH)D_2_, VMR: vitamin D metabolite ratio, g-factor: general cognition.

**Table 2:** Sub-group analyses comparing cognitive function in individuals with deficient and sufficient vitamin D metabolite concentrations.

	**25(OH)D [nmol/L] < 50 (N = 142) versus** **25(OH)D [nmol/L] > 50 (N = 248)**			**24,25(OH)_2_D_3_ [nmol/L] < 3 (N = 124)** **versus** **24,25(OH)_2_D_3_ [nmol/L] > 3 (N = 266)**			**VMR [%] < 3 (N = 54)** **versus** **VMR [%] > 3 (N = 336)**		
	**beta**	**se**	**p**	**beta**	**se**	**p**	**beta**	**se**	**p**
executive function	0.03	0.06	0.68	0.10	0.06	0.09	-0.02	0.09	0.86
visuopractical skills	0.10	0.08	0.20	0.13	0.07	0.07	0.19	0.11	0.07
memory	0.20	0.08	0.01	0.18	0.08	0.02	0.20	0.11	0.07
g-factor	0.12	0.08	0.14	0.16	0.08	0.04	0.24	0.11	0.03

N: number of individuals in analyses, VMR: vitamin D metabolite ratio, β: regression coefficient, SE: standard error of regression coefficient, p: p-value, 25(OH)D: sum of 25(OH)D_3_ + 25(OH)D_2_, g-factor: general cognition.

All analyses are adjusted for age, sex, hypertension, diabetes, atrial fibrillation, education, study, season of blood draw and family structure.

## RESULTS

Subject characteristics, frequency of risk factors and laboratory findings from the total cohort (n = 390) and the two sub-cohorts, ASPS (n = 242) and ASPS-Fam (n = 148) are listed in Table 3. Both cohorts included more females than males. ASPS participants were less educated than ASPS-Fam participants. Hypertension was present in 66.9 % of the ASPS-Fam participants and in 77.3 % of the ASPS participants. The concentrations of the vitamin D metabolites 25(OH)D_3_ and 24,25(OH)_2_D_3_ as well as the VMR were not significantly different between both study cohorts. Only the 25(OH)D_2_ concentration was significantly lower in ASPS than in ASPS-Fam participants (p = < 0.001) (Table 3).

**Table 3:** Cohort baseline characteristics.

**Cohort**	**Total**	**ASPS**	**ASPS-Fam**	p*
**N**	**390**	**242**	**148**	
females, N (%)	241 (61.8%)	151 (62.4%)	90 (60.8%)	0.75
age (years), median [IQR]	69 [63-74]	69 [64 - 75]	68 [55 - 75]	0.002
education (years), median [IQR]	10 [9-13]	10 [9 - 10]	10 [10 - 13]	0.001
hypertension, N (%)	286 (73.3%)	187 (77.3%)	99 (66.9%)	0.24
diabetes, N (%)	49 (12.6%)	31 (12.8%)	18 (12.2%)	0.85
atrial fibrillation, N (%)	23 (5.9%)	14 (5.8%)	9 (6.1%)	0.90
25(OH)D_3_ (nmol/L), median [IQR]	58.8 [39.5 – 76.0]	58.4 [37.9 - 77.9]	59.5 [42.8 - 72.8]	0.76
25(OH)D_2_ (nmol/L), median [IQR]	1.6 [1.0 – 2.5]	1.4 [0.9 - 1.9]	2.2 [1.3 - 3.3]	9.6 x 10^-10^
total 25(OH)D (nmol/L), median [IQR]	60.4 [42.2 – 78.5]	59.7 [40.0 – 79.8]	61.4 [46.1 – 75.2]	0.56
24,25(OH)_2_D_3_ (nmol/L), median [IQR]	3.1 [1.6 – 4.6]	3.1 [1.5 – 4.8]	3.2 [1.9 - 4.5]	0.77
VMR (%), median [IQR]	5.2 [3.9 – 6.8]	5.1 [3.7 - 6.8]	5.4 [4.0 - 6.9]	0.43
blood samples: spring, N (%)	118 (30.3%)	51 (21.2%)	67 (45.3%)	
blood samples: summer, N (%)	110 (28.2%)	76 (31.4%)	34 (23.0%)	
blood samples: autumn, N (%)	87 (22.3%)	66 (28.3%)	21 (14.2%)	
blood samples: winter, N (%)	75 (19.2%)	49 (20.2%)	26 (17.6%)	

ASPS: Austrian Stroke Prevention Study; ASPS-Fam: Austrian Stroke Prevention Family Study; VMR: vitamin D metabolite ratio. *Chi-Square test was used to compare categorical variables and Mann-Whitney-U test was used to compare the non-normally distributed continuous variables between ASPS and ASPS-Fam.

**Figure 2:**
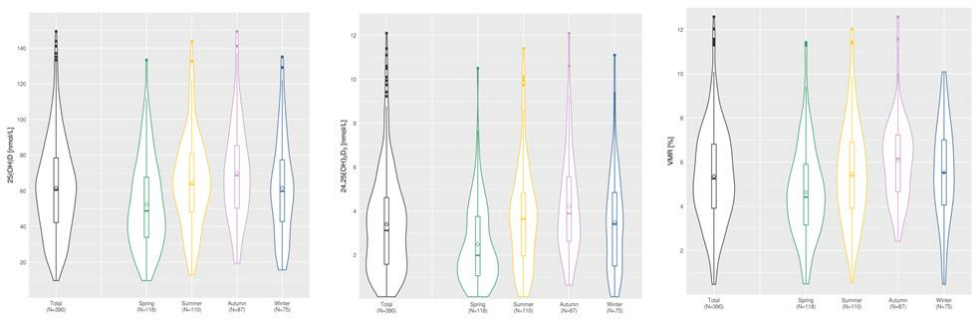
**Seasonal distribution of 25(OH)D, 24,25(OH)_2_D_3_ and VMR in ASPS and ASPS family.** The line within the boxplots denotes the median of the parameter. The crystal within the boxplot denotes the mean of the parameter. ASPS: Austrian Stroke Prevention Study; ASPS-FAM: Austrian Stroke Prevention Family Study; VMR: vitamin D metabolite ratio.

Blood collections were evenly distributed over the year for ASPS, while 45.3 % of the participants from the ASPS-Fam were collected in spring (Table 3). The violin plots in Figure 2 illustrate the seasonal variation of 25(OH)D, 24,25(OH)_2_D_3_ and VMR in ASPS and ASPS-Fam. 25(OH)D, 24,25(OH)_2_D_3_ and the VMR were lower in spring compared to all other seasons (p<0.05) (Table 4).

**Table 4:** Seasonal distribution of vitamin D metabolites.

	**Total Sample (N=390)**	**Spring** **(N=118)**	**Summer** **(N=110)**	**Autumn** **(N=87)**	**Winter** **(N=75)**	p*
25(OH)D (nmol/L), median [IQR]	60.4 [42.2-78.5]	48.8 [33.9-67.6]	63.6 [48.0-80.8]	68.6 [50.2-85.5]	59.8 [42.8-77.3]	< 0.001
24,25(OH)_2_D_3_ (nmol/L), median [IQR]	3.1 [1.6-4.6]	1.9 [1.1-3.8]	3.6 [1.9-4.8]	3.9 [2.6-5.6]	3.4 [1.5-4.9]	< 0.001
VMR (%), median [IQR]	5.3 [3.9-6.8]	4.4 [3.2-5.9]	5.4 [3.9-6.9]	6.2 [4.7-7.2]	5.5 [4.1-7.0]	< 0.001

Spring includes March, April and May; Summer includes June, July and August; Autumn includes September, October, November; Winter includes December, January and February. 25(OH)D: sum of 25(OH)D_3_ + 25(OH)D_2_, VMR: vitamin D metabolite ratio.

*p-value: Mann-Whitney-U test was used to compare Vitamin D metabolites between spring and the other three seasons.

Table 1 shows the linear mixed model analyses between vitamin D metabolites and cognitive function. Total 25(OH)D, 24,25(OH)_2_D_3_ and VMR were significantly related to the test performance on memory. However, after adjustment for multiple testing, none of these associations remained significant. In addition, there was a non-significant association between 24,25(OH)_2_D_3_, VMR and visuopractical skills as well as general cognitive functioning (Table 1). No significant relationships existed between vitamin D metabolites and MRI derived indices of neurodegeneration and vascular changes (Table 5).

**Table 5:** Linear relationship between vitamin D metabolites and MRI in ASPS-Fam.

**ASPS-Fam**	**25(OH)D [nmol/L]**			**24,25(OH)_2_D_3_ [nmol/L]**			**VMR [%]**		
**N = 139**	**β**	**SE**	**p**	**β**	**SE**	**p**	**β**	**SE**	**p**
Total Gray Matter Volume	-5.84E-05	5.70E-05	0.31	4.06E-05	8.78E-04	0.96	1.04E-03	9.69E-04	0.28
Subcortical Gray Matter Volume	-1.18E-05	7.03E-06	0.09	-1.49E-04	1.08E-04	0.17	-1.27E-04	1.22E-04	0.30
Hippocampus Volume	-1.24E-07	7.96E-07	0.88	-9.94E-07	1.22E-05	0.94	-3.30E-06	1.34E-05	0.81
**Cortical Volume**									
Total	-3.00E-05	4.54E-05	0.51	2.52E-04	6.97E-04	0.72	1.03E-03	7.64E-04	0.18
Frontal Lobe	-1.38E-05	1.91E-05	0.47	4.04E-05	2.94E-04	0.89	3.86E-04	3.23E-04	0.23
Temporal Lobe	1.75E-06	1.05E-05	0.87	1.59E-04	1.61E-04	0.32	2.40E-04	1.80E-04	0.18
Parietal Lobe	-1.28E-05	1.31E-05	0.33	1.02E-05	2.01E-04	0.96	2.31E-04	2.21E-04	0.30
Occipital Lobe	-3.08E-06	6.28E-06	0.62	3.70E-05	9.64E-05	0.70	9.33E-05	1.08E-04	0.39
**Cortical Thickness**									
Frontal Lobe	-9.95E-07	3.73E-06	0.79	-1.96E-05	5.74E-05	0.73	-3.80E-05	6.45E-05	0.56
Temporal Lobe	1.28E-06	3.45E-06	0.71	4.42E-05	5.29E-05	0.40	1.97E-05	5.91E-05	0.74
Parietal Lobe	-1.78E-06	3.43E-06	0.60	-1.48E-05	5.27E-05	0.78	-4.16E-06	5.85E-05	0.94
Occipital Lobe	-2.92E-06	2.65E-06	0.27	-2.44E-05	4.08E-05	0.55	-7.66E-06	4.53E-05	0.87
**Cortical Surface Area**									
Frontal Lobe	-3.44E-04	7.14E-04	0.63	5.06E-03	1.10E-02	0.65	2.01E-02	1.23E-02	0.10
Temporal Lobe	-1.43E-04	3.76E-04	0.70	-4.65E-04	5.77E-03	0.94	4.40E-03	6.46E-03	0.50
Parietal Lobe	-2.30E-04	5.24E-04	0.66	2.41E-03	8.05E-03	0.76	9.41E-03	9.03E-03	0.30
Occipital Lobe	2.37E-04	3.03E-04	0.43	4.55E-03	4.65E-03	0.33	3.66E-03	5.24E-03	0.49

ASPS-Fam: Austrian Stroke Prevention Family Study, N: number of individuals in analyses, VMR: vitamin D metabolite ratio, β: regression coefficient, SE: standard error of regression coefficient, p: p-value.

All analyses are adjusted for age, sex, hypertension, diabetes, atrial fibrillation, season at blood draw and family structure. All Freesurfer variables are normalized for total intracranial volume.

Sub-group analyses of individuals with low concentrations of 25(OH)D and 24,25(OH)_2_D_3_ showed significantly worse memory function compared to individuals with normal or high concentrations of these metabolites (Table 2). Correction for multiple testing weakens these associations leaving only a trend (data not shown). No differences in cognitive function were detectable between individuals with a reduced and a normal VMR (Table 2).

## DISCUSSION

The present results suggest a reduced memory function in individuals with low serum concentrations of 25(OH)D and 24,25(OH)_2_D_3_, as well as a low VMR. When considering only 24,25(OH)_2_D_3_ and VMR, similar trends are also detectable for visuopractical skills and global cognitive function. In contrast, none of the vitamin D metabolites is linked to any of the structural indices of neurodegeneration and vascular brain changes.

Several studies have investigated a potential relationship between serum 25(OH)D and cognitive function with mixed results. For example, amongst 2,777 well-functioning, community-dwelling elderly individuals of the Health ABC cohort, serum 25(OH)D was associated with global cognitive function and cognitive decline [45]. Miller et al. described higher rates of decline in episodic memory and executive function in vitamin D deficient individuals, but no association with semantic or visuospatial ability [46]. Additional support that links vitamin D deficiency to cognitive impairment and decline comes from the Dutch Longitudinal Aging Study Amsterdam (LASA), the US Cardiovascular Health Study (CHS) and the ESTHER study [47–50]. However, there have also been contrasting findings. In a rather small study of 64 non- demented, older Portuguese subjects, Carvalho et al. did not find significant cross-sectional or longitudinal correlations between baseline 25(OH)D and composite scores of executive and memory function [51]. The present study expands existing knowledge significantly by combining in depth cognitive testing with advanced MRI analyses of structural brain atrophy and mass spectrometric measurements of vitamin D metabolites in a single cohort of substantial size. Similar to our study, most previous investigations also suggest a decline of cognitive function with decreasing concentrations of 25(OH)D. Nonetheless, previous findings were mainly based on relatively simple screening tools, such as the Mini-Mental State Examination, Trail Making Tests, the digit symbol substitution test (DSST) or a standardized phone interview. In the present study we performed an extensive analysis of all cognitive domains showing a consistent trend that links serum 25(OH)D to memory function. When considering 24,25(OH)_2_D_3_ and VMR, similar relationships were seen for visuopractical skills and global cognitive function as assessed by the g-factor.

It is important to note that previous studies assessed 25(OH)D mainly by immunoassays. These assays are known for their variable analytical performance, which may represent a source of substantial bias [52–54]. None of these assays is capable of detecting additional vitamin D metabolites. Importantly, the simultaneous measurement of 25(OH)D and 24,25(OH)_2_D_3_ as it was done in our study allows to improve the identification of individuals with vitamin D deficiency, CYP24A1 lack of function mutations, vitamin D intoxication or uncontrolled 1,25(OH)_2_D production in granulomatous diseases [22, 55]. Recently the concept of an individual 25(OH)D set-point has been proposed [22]. According to this concept, CYP24A1 is downregulated in the presence of low amounts of 25(OH)D and becomes upregulated when sufficient amounts of active 1,25(OH)_2_D are available. Consequently, in the absence of genetic enzyme defects, detectable amounts of 24,25(OH)_2_D_3_ imply a sufficient supply with vitamin D that allows the maintenance of an adequate vitamin D metabolism. In contrast, undetectable 24,25(OH)_2_D_3_ concentrations suggest functional vitamin D deficiency. This concept is supported by a recent study from Cavalier et al., were in a large cohort of children, adolescents and young adults more than 80 % of individuals with 25(OH)D concentrations between 30 and 50 nmol/L had detectable levels of 24,25(OH)_2_D_3_, which suggests vitamin D sufficiency from a biochemical point of view [22]. In line with this concept, the present study revealed the strongest associations between 24,25(OH)_2_D_3_ and cognitive function. In the linear model 24,25(OH)_2_D_3_ was positively associated with memory, visuopractical skills and global cognitive function. Correction for multiple testing attenuated these effects slightly, but did not abolish the respective trends (p ≤ 0.1). Sub-group analyses comparing individuals with and without low 25(OH)D, 24,25(OH)_2_D_3_ and VMR confirmed the results of the linear model. When considering all results together, there appears to be a consistent pattern that supports a link between serum vitamin D metabolite concentrations, VMR and cognitive function. This approach of interpreting statistical results is in line with a recent publication of Amrhein et al. that question the simple binary interpretation of p-values without considering confidence intervals and patterns of results [56].

Another key finding of the present study is that neither 25(OH)D nor 24,25(OH)_2_D_3_ are related to MRI derived indices of neurodegeneration and vascular changes. In contrast, previous studies reported reduced gray matter volume, lower volumes of hippocampal subfields and connection deficits in 25(OH)D deficient people [57, 12]. In the FRAMINGHAM study, deficient 25(OH)D concentrations were associated with lower hippocampal volumes, but not with total brain volume, white matter hyperintensities, or silent brain infarcts [17].

The absence of a significant relationship between serum 25(OH)D, and 24,25(OH)_2_D_3_ and MRI derived indices of brain atrophy, suggests a functional rather than a structural cause of cognitive impairment in subjects with low vitamin D concentrations. This assumption is supported by studies demonstrating expression of the VDR and 1α-hydroxylase in the hippocampus, hypothalamus, thalamus, cortex, subcortex and substantia nigra, which are related to cognitive function [8, 9]. Observational and experimental evidence suggest that vitamin D deficiency is linked to cholinergic dysfunction in the brain, which may have resulted in worse memory performance in our community-dwelling subjects even in the absence of a neurodegenerative process [12, 13, 58]. A study of Johansson et al. supports this view as it showed an indirect association between lower 25(OH)D and acetylcholinesterase activity in cerebrospinal fluid [13]. Another mechanism that might play a role is the vitamin D-related upregulation of neurotrophic factors, such as NGF, GDNF, and NTF3 [11], which are also known to exert enhancing effects on cognition [5, 59, 60].

Our study has limitations. The percentage of vitamin D deficient participants (25(OH)D <50 nmol/L) is small. This might have reduced the chance of identifying potential associations with cognitive impairment and brain abnormalities in our cohort of community-dwelling persons. Furthermore, the detailed assessment of cognitive function and brain structure resulted in a substantial number of statistical tests requiring correction for multiple testing. In conjunction with the limited number of participants this correction weakened several significances leaving only trends. Recently, scientists and statisticians have proposed to change the traditional approach of interpreting statistical results in a binary fashion on the basis of p-values and to consider confidence intervals and patterns as well [56]. When considering all of our results together, there appears to be a pattern that supports a link between serum vitamin D metabolite concentrations and cognitive function. Another limitation is the lack of information on sun exposure and nutrition, which are potential confounders of vitamin D metabolite concentrations. However, a relatively even distribution of study visits throughout the year and adjustment of all statistical analyses for the time of blood collection accounted for the well-known seasonal variation of 25(OH)D [7]. The limited number of patients with MRI data weakens the statistical power of the present results. Between the study visits of ASPS and ASPS Family, substantial progress in MRI technology and data analyses occurred. Therefore, results from ASPS are not comparable with those from ASPS Family and had to be excluded. Strengths of our study are the extensive diagnostic work up of study participants including a thorough clinical examination, demanding cognitive testing and complete quantitative assessment of focal and global structural brain changes. Another strength is the state-of-the-art measurement of vitamin D metabolites by LC-MS/MS. Sample quality, especially analyte stability, is an important aspect that should be considered when working with samples that have been stored for an extended period of time. In contrast to many other analytes, 25(OH)D and 24,25(OH)_2_D are very stable compounds that change minimally when stored at -80° C frozen [23]. This point is supported by the comparable mean concentrations of 25(OH)D and 24,25(OH)_2_D_3_ in ASPS and ASPS family. The samples of both cohorts have been collected several years apart and despite this fact their mean concentrations were almost identical.

In conclusion, vitamin D deficient individuals appear to have a modest reduction of memory function without structural brain atrophy. As vitamin D deficiency is highly prevalent and effective therapies for cognitive dysfunction and dementia are lacking, future studies should explore if vitamin D supplementation can improve cognitive function.
